# Natural selection among Eurasians at genomic regions associated with HIV-1 control

**DOI:** 10.1186/1471-2148-11-173

**Published:** 2011-06-20

**Authors:** Yann C Klimentidis, Brahim Aissani, Mark D Shriver, David B Allison, Sadeep Shrestha

**Affiliations:** 1 Section on Statistical Genetics, Department of Biostatistics, University of Alabama at Birmingham, Birmingham, AL 35294, USA; 2 Department of Epidemiology, University of Alabama at Birmingham, Birmingham, AL 35294, USA; 3 Department of Anthropology, Pennsylvania State University, University Park, PA 16802, USA

## Abstract

**Background:**

HIV susceptibility and pathogenicity exhibit both interindividual and intergroup variability. The etiology of intergroup variability is still poorly understood, and could be partly linked to genetic differences among racial/ethnic groups. These genetic differences may be traceable to different regimes of natural selection in the 60,000 years since the human radiation out of Africa. Here, we examine population differentiation and haplotype patterns at several loci identified through genome-wide association studies on HIV-1 control, as determined by viral-load setpoint, in European and African-American populations. We use genome-wide data from the Human Genome Diversity Project, consisting of 53 world-wide populations, to compare measures of F_ST _and relative extended haplotype homozygosity (REHH) at these candidate loci to the rest of the respective chromosome.

**Results:**

We find that the Europe-Middle East and Europe-South Asia pairwise F_ST _in the most strongly associated region are elevated compared to most pairwise comparisons with the sub-Saharan African group, which exhibit very low F_ST_. We also find genetic signatures of recent positive selection (higher REHH) at these associated regions among all groups except for sub-Saharan Africans and Native Americans. This pattern is consistent with one in which genetic differentiation, possibly due to diversifying/positive selection, occurred at these loci among Eurasians.

**Conclusions:**

These findings are concordant with those from earlier studies suggesting recent evolutionary change at immunity-related genomic regions among Europeans, and shed light on the potential genetic and evolutionary origin of population differences in HIV-1 control.

## Background

HIV-1 infection is a global health problem and the fourth leading cause of death worldwide [1]. One approach to effective HIV prevention or treatment is to understand the interplay of host molecular mechanisms that are primarily influenced by genetic variation. Multiple genes have been implicated as important genetic determinants of the clinical course of HIV-1 infection by regulating various biological pathways including adaptive and innate immune responses [2]. However, associations with most of these genes appear to be population-specific, and many exhibit marked allele frequency differences between different racial/ethnic groups [3].

This interpopulation genomic variability and these differential associations are still poorly understood, and could plausibly be due to natural selection resulting from distinct environmental or cultural pressures in different geographical populations [3]. For instance, the most promising host genetic variant with biological and functional significance in the control of HIV-1 infection is a 32-bp deletion (*CCR5*-Δ32) in the *CCR5 *gene, which codes for the β-chemokine receptor that the virus uses to enter the host cells [2-5]. The homozygous deletion of a 32-bp segment results in a nonfunctional receptor [6], rendering individuals largely, but not completely, resistant to HIV-1 infection. The frequency of homozygous deletion is < 1.5% among European-descent populations, in which it has been postulated to have arisen in response to immune challenges [5], is rare in African Americans, and completely absent in African and East Asian populations. Studies have also shown evidence of positive selection at other genes involved in HIV-1 pathogenesis, such as *TRIM5α *and *APOBEC3G *[7,8], while others do not find any such evidence [9].

Recent genome-wide association studies (GWAS) in both European Americans (EA) and African Americans (AA) have identified a region within the Human Leukocyte Antigen (HLA) superlocus that is consistently associated with HIV-1 viral load (VL) set-point, and which seems to be the main predictor for disease progression [10-13]. The two single nucleotide polymorphisms (SNPs) with the strongest associations among EA, rs2395029 and rs9264942, are located within 208 kilobases (Kb) of each other in the HLA class I region of chromosome 6, near the *HCP5*, *HLA-B*, and *HLA-C *genes. Among AA, this is also the most strongly implicated region of the HLA [13,14]. Among both AA and EA, several non-HLA loci were also found to be among the 'top hits' (i.e., lowest p-value) in GWAS. In fact, the strongest associations in the GWAS among AA were located outside the HLA region. Thus, if HLA variants implicated in HIV-1 VL control show population differences and evidence of natural selection, one may expect to observe similar patterns at the non-HLA variants that have been implicated in HIV-1 VL control.

While the HIV-1 virus is known to adapt itself in humans with staggering mutation rates, especially in response to therapy, factors such as the very short time since the emergence of the recent HIV-1 epidemic make it unlikely that selection in humans in response to this specific epidemic has exerted a large, detectable effect [15]. It is however possible that these loci were subject to natural selection in the more distant past in response to other pathogens, and this has contributed to the population differences we currently observe in HIV-1 VL control. We therefore hypothesize that the genomic regions most strongly associated with HIV-1 VL control in GWAS have been subject to natural selection and differentiation in some populations. In order to test this hypothesis, we examined patterns of F_ST _and relative extended haplotype homozygosity (REHH) in the genomic regions surrounding both the HLA and non-HLA HIV-1 control-associated loci identified through GWAS in EA and AA. We find evidence of natural selection among Eurasian groups at these loci.

## Results

### F_ST _analysis

**Figure 1:**
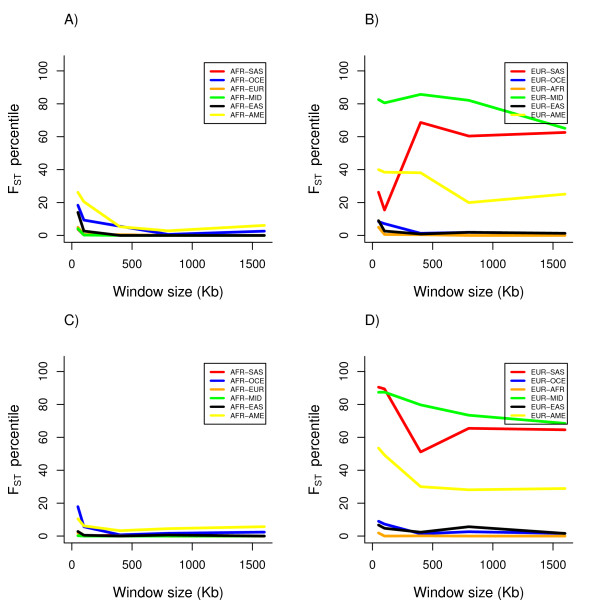
**Pairwise F_ST _percentile in relation to random regions on chromosome 6 at rs2395029 (*HCP5*) with expanding window size (50 Kb to 1600 Kb) for all AFR pairwise comparisons (panel A) and all EUR pairwise comparisons (panel B), and for rs9264942 (*HLA-C*) for all AFR pairwise comparisons (panel C) and all EUR pairwise comparisons (panel D)**. (AFR: sub-Saharan Africa; MID: Middle East; SAS: South Asia; EUR: Europe; EAS: East Asia; OCE: Oceania; AME: Americas)

We first analyzed F_ST _at the loci with the strongest evidence of association among EA (rs2395029 and rs9264942 in the HLA locus; see Table 1). The region surrounding these two loci, which are in low LD (r^2 ^= 0.06, D' = 0.86) in a EA population [11], exhibits very low F_ST _compared to random regions of chromosome 6 for all of the pairwise comparisons with sub-Saharan Africans (AFR), suggesting that much less differentiation than expected has occurred between AFR and other groups (see Figure 1). However, the pairwise comparisons with Europeans (EUR) show that the extent of differentiation between EUR and Middle Easterners (MID) is substantially elevated, as it is between South Asians (SAS) and EUR, compared to the comparisons with AFR. Our bootstrap analysis shows that the F_ST _of AFR-EUR is significantly lower (p < 0.001, and asymptotically, p < 3.8 × 10^-12^) than the F_ST _of MID-EUR, SAS-EUR, and AME-EUR, after considering the chromosomal background differences.

**Table 1:** List of HLA and non-HLA SNPs from the Fellay et al. [11] 'top hits' among European Americans (EA) and the Pelak et al. [13] 'top hits' among African Americans (AA) examined in this study.

	Chromosome	Position in bases	Nearest Gene
**HLA SNPs - EA**			
rs2395029	6	31,539,759	*HCP5*
rs9264942	6	31,332,359	*HLA-C*
rs259919	6	30,133,482	*ZNRD1*
rs9468692	6	30,227,869	*TRIM40*
rs9266409	6	31,444,547	*HLA-B*
rs8192591	6	32,293,774	*NOTCH4*

**HLA SNPs - AA**			
rs2523608	6	31,430,538	*HLA-B*
rs34548063	6	32,047,802	*STK19, DOM3Z*
rs2523933	6	30,040,271	*HLA-A, HCG9*
rs2844538	6	31,456,858	*HLA-B*
rs2596503	6	31,428,789	*HLA-B*

**Non-HLA SNPs - EA**			
rs12185555	2	227,559,317	*IRS1*
rs12557137	X	31,348,807	*DMD*
rs9677779	2	71,903,036	*DYSF*
rs11800642	1	212,909,744	*NSL1*
rs3735118	7	2,944,317	*CARD11*
rs7904001	10	66,762,723	None within 1 Mb
rs16959323	17	10,006,097	*GAS7*
rs5954635	X	141,450,842	*MAGEC2*
rs10073652	5	12,786,980	None within 1 Mb
rs6640729	X	11,346,716	*ARHGAP6*

**Non-HLA SNPs - AA**			
rs454422	20	5,891,693	*MCM8*
rs6948404	7	36,577,018	*AOAH*
rs558718	19	7,815,883	*EVI5L*
rs1357339	11	80,532,023	None within 1 Mb
rs1413191	13	92,011,477	*GPC5*
rs2593321	3	22,097,400	*ZNF659*
rs1116084	8	22,406,934	*PPP3CC*
rs2789066	6	121,141,836	*C6orf170*
rs430374	18	42,499,453	*ST8SIA5*
rs11652146	17	44,774,660	*ZNF652*

**Figure 2:**
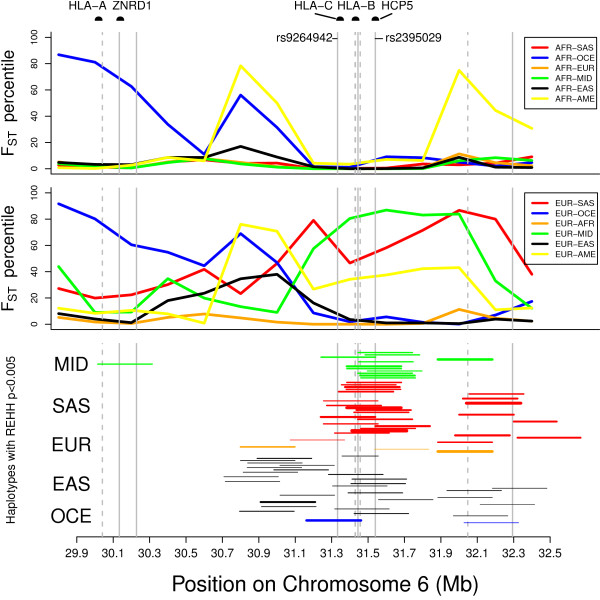
**Sliding window analysis (400 Kb window with 200 Kb overlapping) of F_ST _percentile in relation to random regions on chromosome 6 for all AFR pairwise comparisons (top panel) and for all EUR pairwise comparisons (middle panel)**. The lower panel shows all haplotypes in region with REHH p < 0.005, and core frequency greater than 5%, for each group. Thickness of lines is weighted by core haplotype frequency. AFR and AME are not shown in the lower panel because these groups had no qualifying haplotypes. Loci associated with HIV-1 VL set-point are shown as vertical lines (solid for EA, dotted for AA). Locations of genes of interest are shown in the top of the figure. (AFR: sub-Saharan Africa; MID: Middle East; SAS: South Asia; EUR: Europe; EAS: East Asia; OCE: Oceania; AME: Americas)

**Figure 3:**
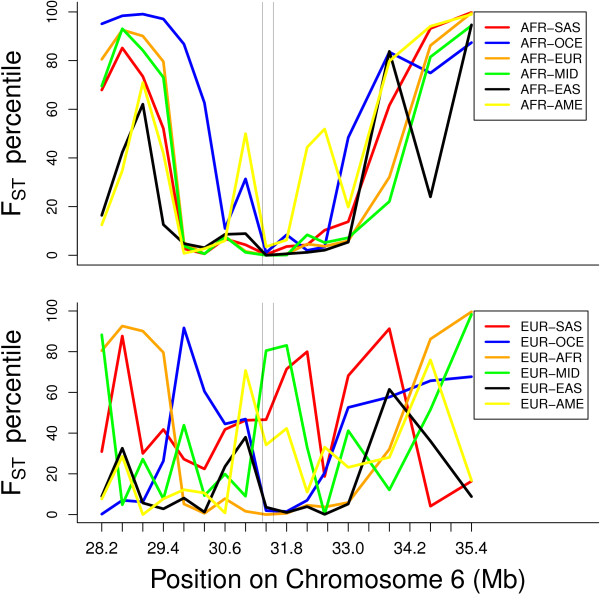
**Sliding window analysis of F_ST _percentile for a wider view of the entire HLA region showing that the low F_ST _for AFR doesn't apply everywhere in the HLA but mainly to the region where the HIV associated loci are 31-32 Mb.** Vertical lines indicate location of rs2395029 and rs9264942. (AFR: sub-Saharan Africa; MID: Middle East; SAS: South Asia; EUR: Europe; EAS: East Asia; OCE: Oceania; AME: Americas)

We next examined whether this pattern of population differentiation applies to the wider region of the HLA that contains four additional independently associated loci among EA, and the top five 'hits' in the HLA among AA (see Table 1, and top two panels in Figure 2). For most of this region we observe a similar pattern of very little differentiation between AFR and other groups and greater differentiation among EUR, MID, and SAS, as well as Oceanians (OCE). Next, we examined the entire HLA region, and find that this pattern of very little differentiation between AFR and other groups applies specifically to the region containing the loci associated with HIV-1 VL control, and is therefore not generalizable to the entire HLA. As shown in Figure 3, this pattern applies approximately to the 31 Mb to 32 Mb region of chromosome 6, which is the region containing variants associated with HIV-1 VL control

**Figure 4:**
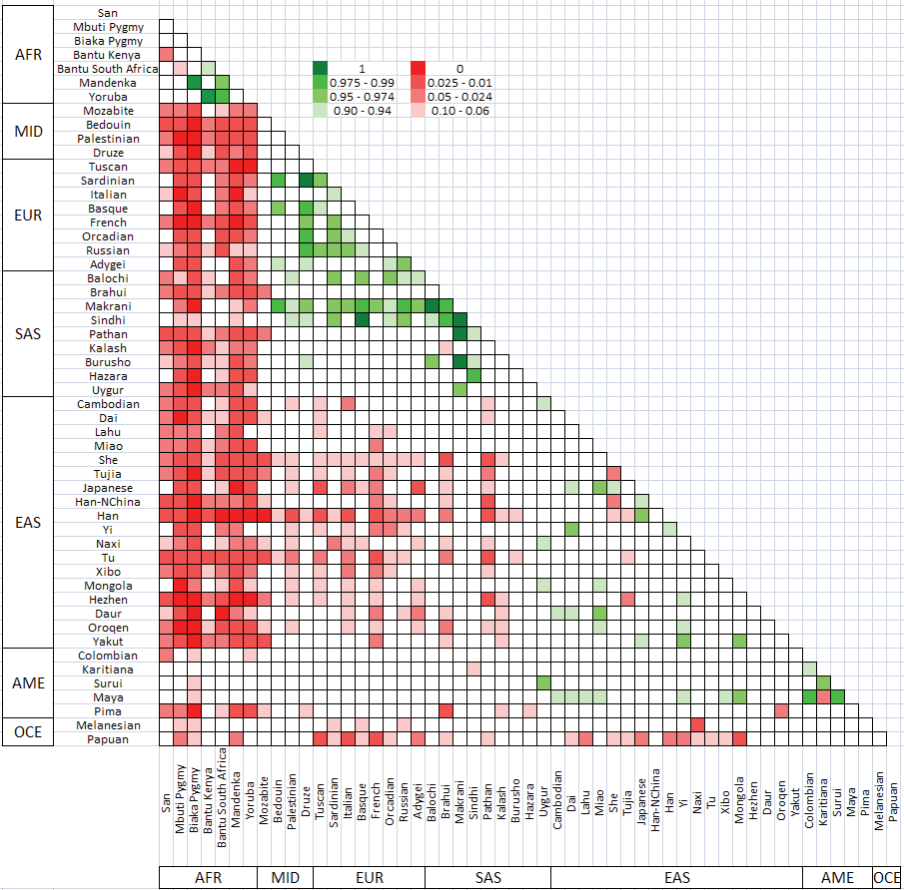
**Pairwise F_ST _matrix for all 53 populations for the 400 Kb region surrounding rs2395029 compared to the rest of the chromosome**. Extremely high F_ST _values from 0.9 to 1.0 are shown in shades of green, where the darkest green represents the 100^th ^percentile and the lightest green represents the 90^th ^- 95^th ^percentile. Extremely low F_ST _values are shown in shades of red, where the darkest red represents the 0^th ^percentile and the lightest red represents the 5^th ^- 10^th ^percentile. (AFR: sub-Saharan Africa; MID: Middle East; SAS: South Asia; EUR: Europe; EAS: East Asia; OCE: Oceania; AME: Americas)

In order to investigate the finer-grained patterns of differentiation among all 53 populations, we focused on the 400 Kb genomic window surrounding rs2395029. As shown in the above analyses, this region exhibits very little differentiation between AFR groups and other groups. However, within AFR, the degree of differentiation appears to be quite elevated among the Bantu, Mandenka, and Yoruba. We observe the greatest degree of differentiation among the Makrani, Sindhi (SAS), and the Druze (MID) populations (see Figure 4).

**Figure 5:**
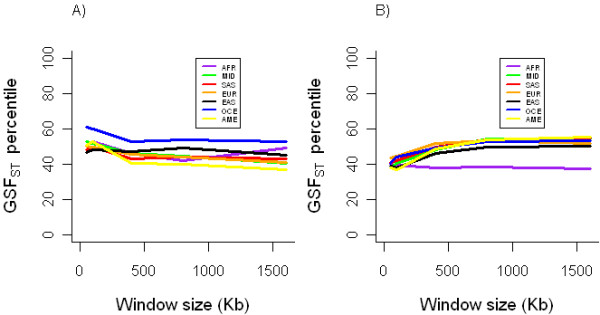
**A) Mean GSF_ST _of top ten non-HLA 'hits' in GWAS among European Americans, and B) Mean GSF_ST _of top ten non-HLA 'hits' in GWAS among African Americans**. A. (AFR: sub-Saharan Africa; MID: Middle East; SAS: South Asia; EUR: Europe; EAS: East Asia; OCE: Oceania; AME: Americas)

F_ST _results for the mean of the top 10 non-HLA 'hits' for each of the EA and AA GWAS (see Table 1) are shown in Figure 5. We find a pattern of differentiation for the AA loci similar to that found for the HLA loci in that the group-specific F_ST _(GSF_ST_) appears to be lower compared to that of other groups. However, this difference is not statistically significant since the 95% CI for the AFR GSF_ST _overlaps with that of other groups. Results for individual loci are shown in Additional Files 1 and 2. Among the AA 'top hits', the region surrounding rs1357339, a SNP in an intergenic region on Chromosome 11, is much less differentiated between AFR and other groups compared to the differentiation among non-AFR. We observe a similar pattern at rs430374 near the *ST8SIA5 *gene on chromosome 18, and at rs9910853 in the *ZNF652 *gene on Chromosome 17. In all of our analyses, we obtained very similar results using cM as opposed to Kb.

### REHH

Depending on population group, among the 27,300 - 42,400 total haplotype cores across chromosome 6, we examined between 346 and 520 haplotype cores in the region delimited by 200 Kb upstream of rs9264942 and 200 Kb downstream of rs2395029. The number of >99^th ^percentile REHH values within this region are shown in Table 2. We find an excess of extreme REHH values among SAS, MID, and East Asians (EAS) compared to AFR and Native Americans (AME). This excess is statistically significant (p < 0.0017) for all pairwise comparisons with AFR and AME, except for EUR and OCE. Q-Q plots (Additional file 3) also show the excess of lower-than-expected p-values among SAS, EAS, and MID, compared to the other groups. We also examined REHH values for haplotypes extending to 150 Kb, and found a similar pattern. We find a similar pattern when using only part of chromosome 6 containing the HLA, instead of the entire chromosome, as the empirical distribution. The lowest panel in Figure 3 shows the location of haplotypes with REHH p-values less than 0.005 for each group, except AFR and AME, for which there are no qualifying haplotypes. Many of these haplotypes are located around rs2395029, thus providing confirmatory evidence (based on both allele frequency differentiation and haplotype length) for recent selection at these loci in the MID, EUR and SAS and EAS groups. We observe an upward trend in F_ST _for the pairwise comparison at the same location where we observe evidence of extreme REHH for a given group. This is especially apparent for MID, SAS, EAS, and EUR. REHH results for the non-HLA 'top hits' are shown in Tables 3 and 4. We find no evidence of major differences among groups in the number of loci exhibiting high REHH values.

**Table 2:** Number of extreme (p < 0.01) REHH values in the 31,112,359 to 31,739,759 base region on chromosome 6 for haplotypes extending to 300 Kb and 150 Kb from core.

Region	AFR	MID	SAS	EUR	EAS	OCE	AME
**Chrom. 6 - 300 Kb**	0	12 (1)	24 (8)	3	21(6)	4	0
**Chrom. 6 - 150 Kb**	1	18 (6)	23 (3)	5 (1)	21 (3)	2	2 (1)
**HLA - 300 Kb**	0	13 (2)	17 (5)	3	8 (1)	2	0

Shown in parentheses are the number of haplotypes with p-value less than 0.001 (>99.9^th ^percentile). Also shown is the REHH when using the HLA region as the empirical distribution as opposed to the entire chromosome. (AFR: sub-Saharan Africa; MID: Middle East; SAS: South Asia; EUR: Europe; EAS: East Asia; OCE: Oceania; AME: Americas)

**Table 3:** Summary of extreme REHH in 200 Kb regions surrounding non-HLA 'top hits' in EA GWAS.

	AFR	MID	SAS	EUR	EAS	OCE	AME	Total groups	Total haplotypes
**rs12185555**	1	1	1	2	0	0	4	5	9
**rs12557137**	0	6	11	1	2	0	0	4	20
**rs9677779**	0	0	2	0	1	1	0	3	4
**rs11800642**	0	0	0	3	2	0	3	3	8
**rs3735118**	0	1	2	0	0	0	3	3	6
**rs7904001**	0	0	0	0	0	0	0	0	0
**rs16959323**	0	1	0	0	2	1	4	4	8
**rs5954635**	1	1	1	3	1	0	0	5	7
**rs10073652**	0	0	0	0	0	0	0	0	0
**rs6640729**	1	2	0	0	0	0	0	2	3

**Total haplotypes**	3	12	17	9	8	2	14		
**Total loci**	3	6	5	4	5	2	4		

Listed is the number of haplotypes with REHH empirical p-value less than 0.01 and core haplotype frequency greater than 5%. (AFR: sub-Saharan Africa; MID: Middle East; SAS: South Asia; EUR: Europe; EAS: East Asia; OCE: Oceania; AME: Americas)

**Table 4:** Summary of extreme REHH in 200 Kb regions surrounding non-HLA 'top hits' in AA GWAS

	AFR	MID	SAS	EUR	EAS	OCE	AME	Total groups	Total haplotypes
**rs12185555**	1	1	1	2	0	0	4	5	9
**rs12557137**	0	6	11	1	2	0	0	4	20
**rs9677779**	0	0	2	0	1	1	0	3	4
**rs11800642**	0	0	0	3	2	0	3	3	8
**rs3735118**	0	1	2	0	0	0	3	3	6
**rs7904001**	0	0	0	0	0	0	0	0	0
**rs16959323**	0	1	0	0	2	1	4	4	8
**rs5954635**	1	1	1	3	1	0	0	5	7
**rs10073652**	0	0	0	0	0	0	0	0	0
**rs6640729**	1	2	0	0	0	0	0	2	3

**Total haplotypes**	3	12	17	9	8	2	14		
**Total loci**	3	6	5	4	5	2	4		

Listed is the number of haplotypes with REHH empirical p-value less than 0.01 and core haplotype frequency greater than 5%. (AFR: sub-Saharan Africa; MID: Middle East; SAS: South Asia; EUR: Europe; EAS: East Asia; OCE: Oceania; AME: Americas)

Finally, we sought to determine whether the susceptibility or the protective allele for rs2395029 and rs9264942 is contained in the core of the haplotypes (with frequency greater than 5%) that are outliers in the REHH distribution (p < 0.05). We find no qualifying haplotypes containing either of these SNPs among AFR and MID. Among SAS, we find that the susceptibility allele (T) for rs2395029 is the allele which is in the core of a haplotype with high REHH (p = 0.0015 and p = 0.0022, in either direction, frequency = 22.5%). This haplotype contains approximately 23% of the susceptibility alleles at this locus present among SAS. Among EUR, the protective allele (G) is in the core of a haplotype with high REHH (p = 0.0029 and p = 0.032 in either direction, frequency = 5.4%). This haplotype contains all protective alleles at this locus present among EUR. Among EAS, the susceptibility allele of rs2395029 is on a high-REHH haplotype (p = 0.0089 and p = 0.046 in either direction, frequency of 18%). This haplotype contains approximately 10% of the susceptibility alleles at this locus present among EAS. The protective allele (C) of rs9264942 is on a high REHH haplotype (p = 0.024, frequency = 9%). This haplotype contains approximately 21% of the protective alleles at this locus present among EAS. Among OCE, the susceptibility allele of rs2395029 is on a high-REHH haplotype (p = 0.028 and p = 0.044, frequency = 30% and 14%, respectively). Since the susceptibility allele is fixed among OCE, these haplotypes contain approximately 30% and 14% respectively, of the susceptibility alleles at this locus among OCE. We find no qualifying haplotypes among AME.

## Discussion

We hypothesized that the genetic variants found to be associated with HIV-1 VL control were subject to recent natural selection and population differentiation. This natural selection could have resulted in the observed population differences in HIV-1 pathogenicity among contemporary populations. We examined the most significant HLA and non-HLA variants associated with HIV-1 VL in GWAS among EA and AA, and found that the top associated loci in the HLA region are located in a sub-region of the HLA that shows very little relative differentiation between the considered sub-Saharan African group and other groups, compared to the relative levels of differentiation among the Eurasian groups considered. We also confirm that the patterns observed in this sub-region are not generalizable across all HLA sub-regions.

Considering all 53 populations, we find the greatest degree of differentiation at the rs2395029 locus for many pairwise comparisons with the Makrani and Sindhi in Pakistan, as well as the Bantu in Africa, and the Druze in Israel. Further studies are needed to confirm and gain a better understanding of the possible reasons for this pattern. Although we take into account a total of 253 SNPs in this locus, these results should be interpreted with some caution given the small sample sizes for some populations.

We find that the differentiation pattern observed in the HLA sub-region also applies to the non-HLA 'top hits', but only among those identified in the HIV-1 VL GWAS among AA. Averaging GSF_ST _over the top ten regions shows a general trend of a smaller degree of differentiation between the sub-Saharan African group and other groups. The lack of differentiation that we observe between the sub-Saharan African and other groups at the HLA sub-region could be due to a high degree of conservation. It is plausible that as each population in Eurasia was subjected to unique selection pressures, a greater degree of differentiation occurred among Eurasian groups than between each of these groups and sub-Saharan Africans. These results should be interpreted with caution because some of the 'top hits' that we examine do not reach genome-wide statistical significance in the respective GWAS.

Paralleling the pattern in differentiation, we observed evidence of extreme relative extended haplotype homozygosity (REHH) among Eurasian groups but not among sub-Saharan Africans or Native Americans. On the basis of our finding of multiple haplotypes with extreme REHH and at relatively low frequencies (i.e. all below 37%), our results appear to be more consistent with a mode of recent evolution characterized by multiple soft sweeps as opposed to single hard sweeps [16].They also suggest that the patterns of differentiation and REHH are not uniform and homogenous across all Eurasians. Instead, it is possible that population-specific patterns of genetic change, perhaps in response to region-specific selection pressures, resulted in unique localized adaptations in different Eurasian populations. Our REHH results suggest that the evidence for selection in the HLA among Eurasian groups is one in which several different haplotypes of low to moderate frequency have spread through populations. In this context, it should be noted that REHH may not be powerful enough to detect very low frequency haplotypes [17]. Other tests of selection such as XP-EHH (using the HGDP browser: http://hgdp.uchicago.edu/cgi-bin/gbrowse/HGDP/) do not appear to show similar trends as those we obtained using F_ST _and REHH. This may be due to the fact that XP-EHH is most powerful for cases of selection in which the selected haplotype reaches a very high frequency in one population but not another, which does not appear to be the situation for the loci that we have examined.

Among Europeans in the HGDP, all rs2395029 protective alleles (G) are on a haplotype that has high REHH, suggesting that this allele or one linked to it may have quickly arisen in response to a selective pressure such as an infectious disease in Europe. However, among South Asians, East Asians and Oceanians, it is the susceptibility allele at this SNP that is the allele present on the haplotype that has high REHH. The inconsistency of these results could signify, among several things, that there are many different polymorphisms in this region that could have functional significance, that the genetic basis of adaptation to similar pressures could be different among different groups, and finally, that the genetic basis of HIV-1 control could be different among different racial/ethnic groups.

Our findings are consistent with recent findings that signatures of selection among Europeans are enriched for immunity related genes in the HLA [18,19]. In fact, Kudaravalli et al. [20] recently found that SNPs associated with gene expression levels of *HLA-C *also show evidence of selection in both Europeans and East Asians in the HapMap samples. Along with these and other studies (see [21]), our findings suggest the presence of selection pressures on the immune system, possibly due to geographic, demographic, cultural, or environmental factors related to subsistence. For example, our fine-scaled analysis of all 53 HGDP populations shows that among the sub-Saharan populations, those populations that show elevated F_ST _between each other (Bantu, Mandenka, and Yoruba) are also the groups that have a history of practicing agriculture. It should be noted that we have not examined populations from East Africa, as these may show very unique patterns, especially considering that the frequency of the rs2395029 protective allele among the Maasai in the HapMap3 sample is quite elevated (14%), and that Henn et al. have found that this allele is also at a relatively high frequency among the Sandawe (17%) in East Africa, and that it exhibits evidence of recent selection in this group [22].

While we have found evidence suggesting the action of natural selection on the genomic regions associated with HIV-1 VL among Eurasian populations, one has to be cautious in interpreting our results. Although in all of our analyses, we have controlled for the genomic background by comparing F_ST _and REHH at the locus of interest to a distribution of other loci on the respective chromosome, we have not necessarily directly tested whether the patterns we observe are consistent with natural selection as opposed to more stochastic evolutionary forces such as genetic drift. Although it is difficult to distinguish selection from drift with certainty, the greater extended haplotype homozygosity among Eurasians coinciding with elevated differentiation among these groups makes the situation we have outlined (i.e. positive selection among Eurasians and balancing selection among some sub-Saharan Africans) more compelling.

## Conclusions

In conclusion, our results show that the HLA region, as well as some of the non-HLA regions, that contain loci associated with HIV-1 VL control among persons of both European and African ancestry, appear to have been subject to natural selection, resulting in population differentiation among Eurasian groups, but not among other groups. Future studies on different samples and/or with different methods will be needed to confirm our findings, and should examine more fine-scale patterns both at the geographic and genetic level. Understanding the genome-wide selection pressure on HIV-1-interacting proteins can provide insights into the evolutionary dynamics of host factors, the genetic basis of differences between nonpathogenic and pathogenic lentivirus infection, and the roles of individual genes in host-pathogen interaction and immunopathogenesis.

## Methods

### HGDP populations and genotypes

We used the publicly available data from the Human Genome Diversity Project (HGDP) collection of 938 individuals from 53 different populations, genotyped at over 650,000 SNPs on the Illumina 650Y platform, and phased using fastPHASE [23]. Following recent reports [24,25] we grouped the 53 populations into seven broader groups based on geographical regions: Sub-Saharan Africa (AFR; n = 102), Middle East (MID; n = 160), South Asia (SAS; n = 200), Europe (EUR; n = 156), East Asia (EAS; n = 229), Oceania (OCE; n = 28), and America (AME; n = 63). A listing of the populations in each group can be found in Additional file 4.

### HIV-1-associated loci from GWAS studies

The set of loci examined in this study (see Table 1) was determined from two HIV-1 GWAS, one performed in European Americans (EA) [11], and the other in African Americans (AA) [13]. In EA, the most strongly associated variants were located in the HLA region of chromosome 6. In AA, the most strongly associated variants were located in various parts of the genome, including the same HLA region found in EA. We chose the top 2 'hits' (according to p-value) in the EA study (rs2395029, rs9264942). We also examined four other independent associations in the HLA region (rs259919, rs9468692, rs9266409, rs8192591) reported in the EA study, and the top five HLA 'hits' in the AA study. We also examined the top ten non-HLA 'hits' in each of the two studies. Among these top ten 'hits', we only considered the SNP with the lowest p-value if more than one in the same genomic region made the list.

### F_ST_

The F_ST _statistic partitions the total genetic variance into within- and between- population components, thereby quantifying the extent of population differentiation [26]. An elevated F_ST _at a given locus relative to the rest of the genome indicates a high degree of differentiation among populations, which may be indicative of positive selection, while a low F_ST _is consistent with balancing or purifying selection, for example in highly conserved regions of the genome. Since individual SNP-based estimates of F_ST _are highly variable and may therefore be unreliable indicators of differentiation at a genomic locus [27,28], we calculated an average of F_ST _values for all SNPs contained in varying window sizes, from 50 Kb to 1.6 Mb ("zooming out" by a factor of two), each centered on the HIV-1-associated SNP. Given that we are interested in relatively recent selection events, a window-based estimate of F_ST _allows us to detect the hitchhiking of loci near the putatively selected variants. In addition, a window-based approach to F_ST _seems preferable since an associated variant detected by GWAS may not represent the actual causal variant, but instead a proxy for a nearby variant. In order to account for variation in recombination patterns along the genome, we also perform the F_ST _analyses using cM distance instead of Kb distance. We used the hg18/build 36 genetic map, retrieved from http://hgdp.uchicago.edu/Browser_tracks/Genetic_Maps/.

To calculate F_ST_, we used the method of Weir and Cockerham [29]. We calculated a single global estimate of F_ST _(based on all 7 population groups), as well as all 21 pairwise estimates of F_ST_. F_ST _was not calculated for a SNP if that SNP is monomorphic in the groups being compared. Negative values of F_ST _were given a value of 0 because negative values are biologically meaningless.

To control for population-specific demographic effects on the genome, we compared the F_ST _in the risk window to a null distribution of F_ST _values calculated in randomly chosen windows. Specifically, for each risk SNP, we randomly chose 1000 equally sized (in bp) windows along the same chromosome, with similar genic/non-genic content (± 10%), since F_ST _tends to be slightly higher for genic SNPs [30]. The genic/non-genic classification was performed according to the annotation provided by Sullivan et al. (https://slep.unc.edu/evidence/?tab=Downloads), which classifies a SNP based on whether it is in the transcribed region of a gene. SNP annotations were created using the TAMAL database [31], based chiefly on UCSC genome browser files [32], HapMap [33], and dbSNP [34].

For all 21 possible pairwise F_ST _comparisons, we obtained percentile ranks of the window centered on the risk SNP, compared to the 1000 randomly centered windows across chromosome 6, and use these to compare differentiation of the risk-SNP window across groups. However, in order to determine the significance of the difference between the risk-SNP window F_ST _between populations A and B and the same risk-SNP window F_ST _between populations A and C, while considering the difference in null chromosomal background (through the mean F_ST _of 1000 randomly chosen windows, excluding the risk-SNP window, as described above), we generated 1000 bootstrap resamples from the total sample of 938 individuals. For each of the bootstrap resamples we calculated the risk-SNP window F_ST _and the mean F_ST _of the 1000 random (non-risk) windows. We then tested the null hypothesis that the difference in risk-SNP window F_ST _is the same as the difference for the mean of the random windows, formulated as follows:

where (θ_X, A-B_) is the F_ST _for the risk window (X) between population A and B, θ_X, A-C _is the F_ST _for the risk window (X) between population A and C,  is the F_ST _at regions other than the risk region X () between population A and B, and  is the F_ST _at regions other than the risk region X () between population A and C. To do so, we assumed that the sample estimators of the four θ's utilized in the null hypothesis stated above are asymptotically unbiased estimators. Let us denote the sample estimators of the population parameters by the use of a circumflex and let: . Under the null hypothesis and the assumption that the estimators are unbiased, E(*T*) = 0. Therefore, testing the null hypothesis that E(*T*) = 0 is a valid test of the null stated above. In an ordinary sample of independent observations (individuals) taken from the populations of interest, one can test E(*T*) = 0 using bootstrap methods. None of the above analyses depend on any of the s or any quantities on which they are based being independent. The value of *T *from the observed sample *T*_*obs *_was calculated. One thousand bootstrap samples were taken from the original sample with replacement, and a value of *T *calculated in each bootstrap sample, denoted  for the *i*th bootstrap sample. A p-value to test the null hypothesis stated above was then calculated in two ways, conservatively as , and asymptotically by letting  be the ordinary sample standard deviation of the T*, and assuming that the  is asymptotically distributed as χ^2 ^with 1 df. Due to computational limitations, we restricted this analysis to the rs2395029 SNP.

Given that we are interested in examining the top ten non-HLA variants as a set, as well as each one separately, we averaged the 10 percentiles of all pairwise F_ST _comparisons. In order to obtain a group-specific F_ST _for a given group, which we refer to as GSF_ST_, we averaged the F_ST _percentile ranks of the six pairwise comparisons that contain the group in question. For example, to obtain an estimate of GSF_ST _for AFR, we took the average of the percentile F_ST _of the following six pairwise comparisons: AFR-MID, AFR-SAS, AFR-EUR, AFR-EAS, AFR-OCE, and AFR-AME. Instead of considering many pairwise comparisons for each locus, GSF_ST _allows us to examine how differentiated a given group is at a given locus compared to all other groups, and relative to the rest of the chromosome.

Because the associated loci in the HLA region were clustered in a relatively small region, we examined the mean F_ST _of all SNPs in a sliding 400 Kb window with an overlap of 200 Kb, from 29.8 to 32.5 Mb on chromosome 6. We also examined the entire HLA region to determine if the pattern observed in the 29.8 to 32.5 Mb region is characteristic of the HLA region as a whole (28 to 35.6 Mb). To investigate the level of differentiation among all 53 populations, we constructed a matrix of F_ST _percentiles for all possible pairwise comparisons for the 400 Kb region surrounding rs2395029.

### REHH

Extended haplotype homozygosity (EHH) is defined as the probability that two randomly chosen chromosomes carrying the core haplotype of interest are identical by descent, and the relative EHH (REHH) is the factor by which EHH decays on the tested core haplotype compared to that of other core haplotypes combined [35]. The REHH thus corrects for local variation in recombination rates. We obtained REHH values using Sweep software v1.1 [35], (downloaded from http://www.broadinstitute.org/mpg/sweep). For all associated SNPs, we used the same phased haplotype data as above, and examined REHH at haplotypes containing the HIV-1 risk SNP, and all haplotypes contained in the surrounding 400 Kb region (200 Kb in either direction). Core haplotypes were defined according to the definition of a haplotype block in Gabriel et al. [36], and REHH was measured 300 Kb and 150 Kb away from the core haplotype. For each region and each population group, we compared the REHH in the risk SNP region to the entire chromosome on which the region resides, to determine if the candidate region contains haplotypes with exceptionally high REHH, binning by haplotype frequency. We only considered core haplotypes with frequency greater than 5%.

We examined REHH in the region surrounding both the 'top hits' in the EA and AA study, and 200 Kb outward in either direction, and noted all instances in which a haplotype had a REHH value in the top 99.9^th^, 99.5^th^, 99^th^, and 95^th ^percentile of the empirical distribution, binning by haplotype frequency. We use Q-Q plots to examine the distribution of observed vs. expected -log_10 _p-values for the REHH values in this region, for each of the population groups. For the wider HLA region that encompasses other GWAS top 'hits', we considered all haplotypes from 29.8 to 32.5 Mb on chromosome 6. We use the entire chromosome 6, as well as the entire HLA region as empirical distributions. We used a two-sided Fisher's exact test to determine whether there is an excess of instances of extreme REHH in one or more groups.

## Authors' contributions

SS and YCK developed the concept behind the study. YCK, SS, and BA designed the experiment and carried it out; YCK designed and carried out the data analysis with help from BA, MDS, and DBA; YCK and SS wrote the paper. BA, MDS, and DBA provided critical reviews of the manuscript. All authors have read and approved the final manuscript.

## Supplementary Material

Additional file 1 **GSF_ST _percentile for top ten non-HLA 'hits' among African Americans**. Window size (Kb) is on the x-axis while GSF_ST _percentile in on the y-axis, for windows centered on the top ten non-HLA 'hits' in the African-American GWAS. Click here for file

Additional file 2 **GSF_ST _percentile for top ten non-HLA 'hits' among European Americans**. Window size (Kb) is on the x-axis while GSF_ST _percentile in on the y-axis, for windows centered on the top ten non-HLA 'hits' in the European-American GWAS. Click here for file

Additional file 3 **Q-Q plots of REHH p-values by group**. Observed vs. expected -log_10 _p-values for REHH in seven groups, considered in the 31.1. to 37.3 Mb region of chromosome 6. P-values are empirical, based on the distribution of all REHH p-values for all of chromosome 6. Click here for file

Additional File 4 **Details of HGDP sample**. A listing of the 53 populations in the HGDP sample and how they were grouped. Click here for file
