# Supplementary material for: Natural selection among Eurasians at genomic regions associated with HIV-1 control
Source: BMC Evol Biol. 2011 Jun 20;11:173. doi: 10.1186/1471-2148-11-173 (PMC3141432; doi:10.1186/1471-2148-11-173)
Supplement: Additional file 1 — GSFST percentile for top ten non-HLA 'hits' among African Americans. Window size (Kb) is on the x-axis while GSFST percentile in on the y-axis, for windows centered on the top ten non-HLA 'hits' in the African-American GWAS. [file 1471-2148-11-173-S1.DOCX]

**Additional file 1**

**Title:** GSF_ST_ percentile for top ten non-HLA ‘hits’ among African Americans.

**Description:** Window size (Kb) is on the x-axis while GSF_ST_ percentile in on the y-axis, for windows centered on the top ten non-HLA ‘hits’ in the African-American GWAS.

rs454422, Chr. 20, MCM8

rs6948404, Chr. 7, AOAH

rs558718, Chr. 19, EVI5L

rs1357339, Chr. 11, intergenic

rs1413191, Chr. 13, GPC5

rs2593321, Chr. 3, AC023798.16

rs4872511, Chr. 8, PPP3CC

rs2789066, Chr. 6, RP11 100A16.1

rs430374, Chr. 18, ST8SIA5

rs9910853, Chr. 17, ZNF652
