# Supplementary material for: Natural selection among Eurasians at genomic regions associated with HIV-1 control
Source: BMC Evol Biol. 2011 Jun 20;11:173. doi: 10.1186/1471-2148-11-173 (PMC3141432; doi:10.1186/1471-2148-11-173)
Supplement: Additional file 2 — GSFST percentile for top ten non-HLA 'hits' among European Americans. Window size (Kb) is on the x-axis while GSFST percentile in on the y-axis, for windows centered on the top ten non-HLA 'hits' in the European-American GWAS. [file 1471-2148-11-173-S2.DOCX]

**Additional file 2**

**Title:** GSF_ST_ percentile for top ten non-HLA ‘hits’ among European Americans.

**Description:** Window size (Kb) is on the x-axis while GSF_ST_ percentile in on the y-axis, for windows centered on the top ten non-HLA ‘hits’ in the European-American GWAS**.**

rs12185555, Chr. 2, intergenic

rs12557137, Chr. X, intergenic

rs9677779, Chr. 2, DYSF

rs11800642, Chr. 1, NSL1

rs3735118, Chr. 7, CARD11

rs7904001, Chr. 10, intergenic

rs16959323, Chr. 17, GAS7

rs5954635, Chr. X, intergenic

rs10073652, Chr. 5, intergenic

rs6640729, Chr. X, ARHGAP6
