# Supplementary material for: Natural selection among Eurasians at genomic regions associated with HIV-1 control
Source: BMC Evol Biol. 2011 Jun 20;11:173. doi: 10.1186/1471-2148-11-173 (PMC3141432; doi:10.1186/1471-2148-11-173)

**Additional file 3**

**Title:** Q-Q plots of REHH p-values by group.

**Description:** Observed vs. expected –log_10_ p-values for REHH in seven groups, considered in the 31.1. to 37.3 Mb region of chromosome 6. P-values are empirical, based on the distribution of all REHH p-values for all of chromosome 6.


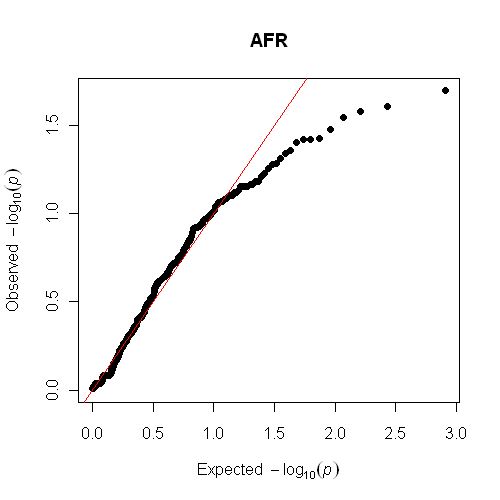

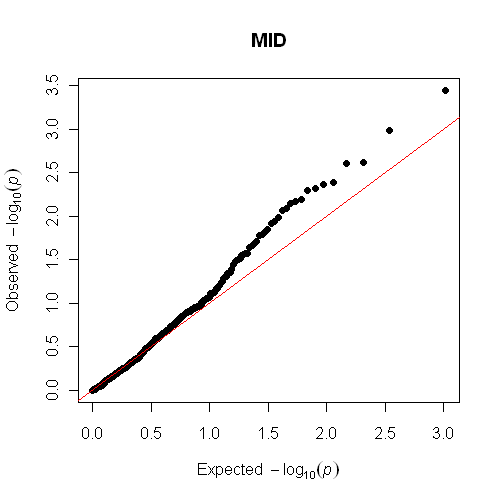

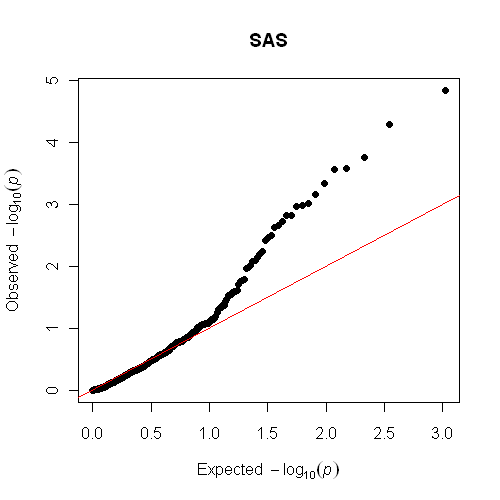


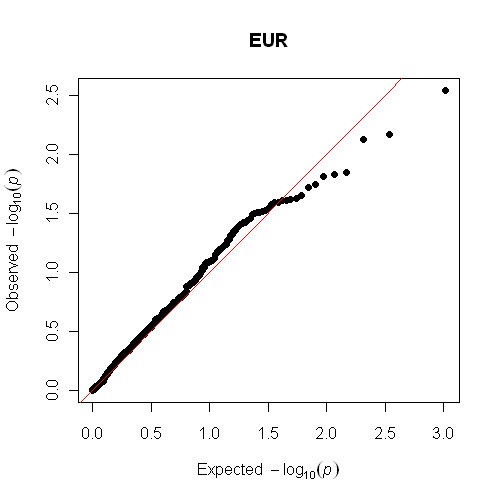

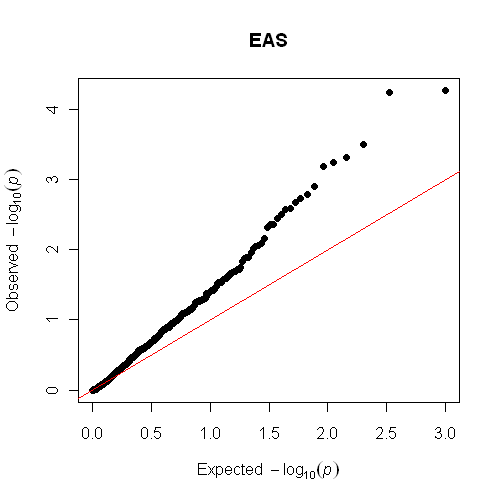

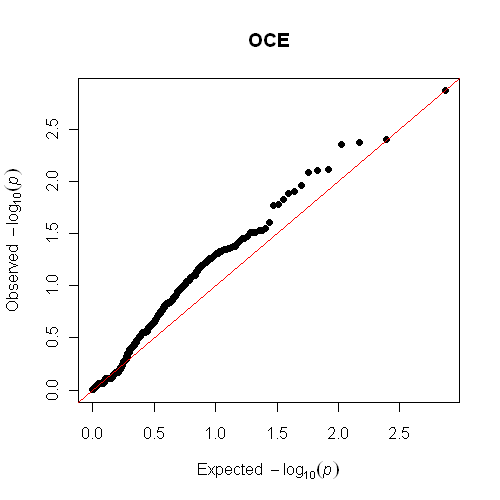


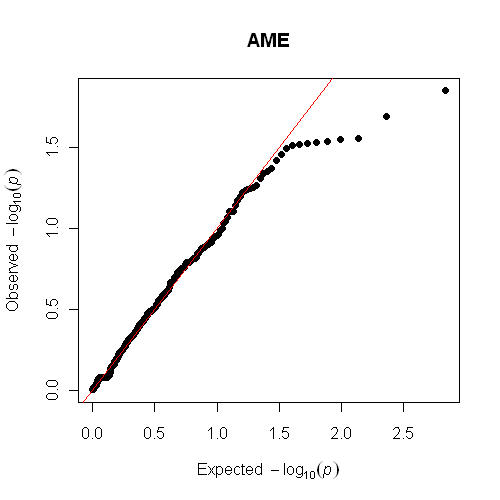

Supplement: Additional file 3 — Q-Q plots of REHH p-values by group. Observed vs. expected -log10 p-values for REHH in seven groups, considered in the 31.1. to 37.3 Mb region of chromosome 6. P-values are empirical, based on the distribution of all REHH p-values for all of chromosome 6. [file 1471-2148-11-173-S3.DOCX]
