# Supplementary material for: Natural selection among Eurasians at genomic regions associated with HIV-1 control
Source: BMC Evol Biol. 2011 Jun 20;11:173. doi: 10.1186/1471-2148-11-173 (PMC3141432; doi:10.1186/1471-2148-11-173)
Supplement: Additional File 4 — Details of HGDP sample. A listing of the 53 populations in the HGDP sample and how they were grouped. [file 1471-2148-11-173-S4.DOCX]

**Additional File 4**

**Title:** Details of HGDP sample

**Description:** A listing of the 53 populations in the HGDP sample and how they were grouped.

The Sub-Saharan African group consists of South African and Kenyan Bantu, San and Yoruba from Nigeria, Mandenka from Senegal, Biaka pygmy from the Central African Republic, and Mbuti pygmy from the Democratic Republic of Congo. The Middle East group consists of Mozabite from Algeria, and Bedouin, Druze and Palestinian from Israel. The South Asian group consists of Brahui, Balochi, Hazara, Makrani, Sindhi, Pathan, Kalash, and Burusho, all from Pakistan. The European group consists of French, Basques, Italians, Orcadians, Adygei and Russians. The East Asian group consists of Han and other Chinese ethnic groups, Yakut from Siberia, Japanese and Cambodian. The Oceania group consists of Papuan from New Guinea and Melanesian from Bougainville. The American group consists of Pima and Maya from Mexico, Piapoco and Curripaco from Colombia, and Karitiana and Surui from Brazil.
